# Tuning the Schottky barrier height in single- and bi-layer graphene-inserted MoS_2_/metal contacts

**DOI:** 10.1038/s41598-024-67150-2

**Published:** 2024-09-08

**Authors:** Xumei Zhao, Caijuan Xia, Lianbi Li, Anxiang Wang, Dezhong Cao, Baiyu Zhang, Qinglong Fang

**Affiliations:** 1 School of Science, Xi’an Polytechnic University, Xi’an, 710048 Shaanxi China; 2 Materials Department, University of California, Santa Barbara, CA 93106-5050 USA

**Keywords:** MoS_2_, Electronic properties, Schottky barrier, First-principle calculations, Materials science, Nanoscience and technology, Physics

## Abstract

First-principle calculations based on density functional theory are employed to investigate the impact of graphene insertion on the electronic properties and Schottky barrier of MoS_2_/metals (Mg, Al, In, Cu, Ag, Au, Pd, Ti, and Sc) without deteriorating the intrinsic properties of the MoS_2_ layer. The results reveal that the charge transfer mainly occurs at the interface between the graphene and metal layers, with smaller transfer at the interface between bi-layer garphene or between graphene and MoS_2_. And the tunneling barrier exists at the interface between graphene and MoS_2_, which hinders electron injection from graphene to MoS_2_. Importantly, the Schottky barrier height ($\Phi_{{\text{SB,N}}}$) decreases upon graphene insertion into MoS_2_/metal contacts. Specifically, for single-layer graphene, the $\Phi_{{\text{SB,N}}}$ of MoS_2_ contacted with Mg, In, Sc, and Ti are − 0.116 eV, − 0.116 eV, − 0.014 eV, and − 0.116 eV, respectively. Furthermore, with bilayer graphene, when by inserting bi-layer graphene, the negative n-type Schottky barrier of − 0.086 eV, − 0.114 eV, − 0.059 eV, − 0.008 eV, and − 0.0636 eV are observed for MoS_2_ contacted with the respective metals, respectively. These findings provide a practical guidance for developing and designing high-performance transition metal dichalcogenide nanoelectronic devices.

## Introduction

Two-dimensional (2D) transition metal dichalcogenides (TMDs) have attracted considerable attention as potential channel materials for next-generation nanoelectronic devices due to their atomic thickness, high carrier mobility, low concentration of surface dangling bonds, and suitable band gap^1–3^. Previous studies on 2D MoS_2_ transistors have demonstrated excellent field-effect mobility with high on–off ratio at room temperature^4–6^. However, due to the Fermi level pinning, the contact resistance of MoS_2_/metal contact is up to 5 kΩ·μm–1 MΩ·μm, which is more than 30 times larger than that of the Si/metal^7,8^. This high contact resistance poses a significant barrier to realizing the low power application potential of MoS_2_-based devices.

The origin of high contact resistance for MoS_2_/metal remains unclear, even though several factors have been proposed, including the wide contact tunnel barrier, the high Schottky barrier ($\Phi_{{{\text{SB}}}}$), and high intrinsic resistance of semiconductor channel^9–11^. In principle, the transport properties of 2D materials devices are often limited by the contact tunnel barrier and Schottky barrier rather than the intrinsic resistance. Efforts have been made to mitigate Schottky barrier by employing low work function metals. For instance, Das et al.^4^ achieved enhanced effective mobilities by using scandium contacts on exfoliated MoS_2_ flakes covered by a 15 nm Al_2_O_3_ film. However, to date, the reported lowest contact resistance using scandium electrode is still far from satisfactory. Novel doping strategies for TMDs have also been explored to reduce the Schottky barrier^12–15^. However, the reliable doping technology with precise control over doping concentration and doping profile for 2D transistors^16^. Recent experiments have demonstrated significant Schottky barrier reduction when ultrathin tunnel layer is inserted between MoS_2_ and metal electrode^17–22^. For example, through a dry transfer technique and a metal-catalyzed graphene treatment process, Leong et al.^23^ fabricated nickel-etched-graphene electrodes on MoS_2_ that yield contact resistance as low as 200 Ω·μm. Du et al.^24^ claimed that MoS_2_/graphene/Ti Schottky barrier provides electron injection efficiency up to 130 times higher in the subthreshold regime when compared with MoS_2_/Ti, which resulted in V_DS_ polarity dependence of device parameters such as threshold voltage (V_TH_) and subthreshold swing (SS). Chanana et al.^25^ proposed that unlike MoS_2_/metal contacts, the projected dispersion of MoS_2_ remains preserved in MoS_2_/graphene/metal contacts with shift in the bands on the energy axis. A proper choice of metal may exhibit ohmic nature in a graphene-inserted MoS_2_/metal contact. Moreover, Qiu et al.^26^ demonstrated that the contacts of the multi-layered MoS_2_/graphene have tunable negative barriers in the range of 300 to − 46 meV as a function of gate voltage. Thus, 2D materials insertion shows great potential to effectively adjust the contact properties of MoS_2_/metal contacts.

Graphene has been identified as an effective approach to adjust the work function of the metal^27^. it interacts strongly with the metals, such as Co, Ni, Pd and Ti, which involves hybridization between graphene *p*_z_ states and metal *d* states and reduces considerably the work function of the metal^28^. Moreover, the substantial potential drop (8.881 eV) between MoS_2_ and graphene could promote the charge transfer from graphene to MoS_2_ layer. In this work, we investigate the electronic properties and Schottky barrier of MoS_2_/metals (Mg, Al, In, Cu, Ag, Au, Pd, Sc, and Ti) by inserting single- and bi-layer graphene based on density functional theory (DFT). Our study reveals that the tunneling barrier existed at the interface between graphene and MoS_2_, which hinders electron injection from graphene to MoS_2_. We achieved the decrease of $\Phi_{{\text{SB,N}}}$ upon graphene insertion in MoS_2_/metal contacts. The results are systematically discussed, and provide valuable insights for design of high-performance device.

## Computational details

First-principle calculations based on DFT were carried out by using the Vienna ab initio simulation package (VASP)^29,30^. The projector augmented wave (PAW) method^31^ was used to describe the electron–ion core interaction, which is more accurate than the ultra-soft pseudo-potentials. The Perdew-Burke-Ernzerhof (PBE)^32^ formulation of the generalized gradient approximation (GGA) was chosen to describe the exchange–correlation interaction. Since the semi-local functional, such as, GGA fail to describe weakly interacting systems, the van der Waals interaction in the Grimme approach was adopted to describe the weak interlayer interaction^33^. The cutoff energy for the plane-waves was chosen to be 450 eV. The Brillouin-zone integration was performed by using an 11 × 11 × 1 k-mesh according to the Monkhorst–Pack scheme and Gaussian smearing broadening of 0.05 eV was adopted. To avoid artificial interactions between the periodic images of the structures, a vacuum region of at least 15 Å was used. A conjugate-gradient algorithm was employed to relax the ions to the ground states with an energy convergence of 1.0 × 10^–5^ eV and a force convergence of 0.02 eV/Å on each ion, respectively. Visualizations of supercell and structure were done with the software VESTA^34^.

## Results and discussions

**Figure 1:**
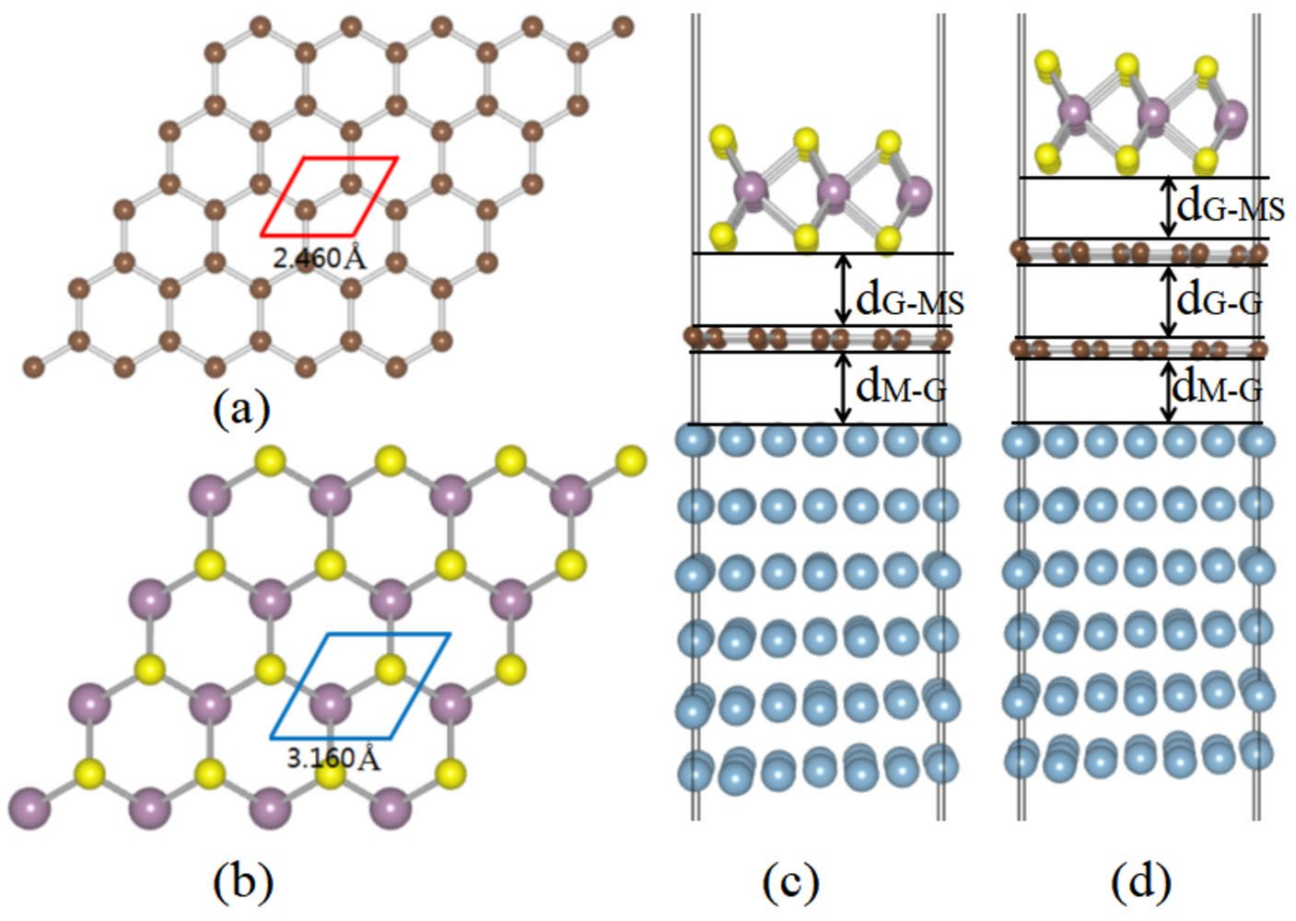
Optimized geometries for top view of monolayers (**a**) graphene and (**b**) MoS_2_. Side view of MoS_2_/metals by inserting graphene: (**c**) single-layer and (**d**) bi-layer.

The optimized structures of single-layer graphene and MoS_2_ are shown in Fig. 1a,b, with the lattice constants of 2.460 Å and 3.160 Å, respectively, which is consistent with previous experimental and theoretical results.^35,36^ In the optimized configurations, the bond lengths of C–C and Mo–S are 1.406 and 2.413 Å, respectively. Figure 1c illustrates the slab of the stacking MoS_2_/graphene/metal contact configuration, containing six layers of metal, single-layer graphene and MoS_2_. A vacuum of 15 Å in thickness is included to avoid spurious interactions. Due to the the different lattice constants of the components, the supercells of the coincidence site lattice (CSL) in the commensurable structures are commonly different. The in-plane supercells are constructed to minimize the lattice misfit strain between MoS_2_, graphene, and metal, in which a basis vector on a given metal surface is denoted by $h_{1} \mathop c\limits^{ \to }_{1} + h_{2} \mathop c\limits^{ \to }_{2}$, with ($\mathop c\limits^{ \to }_{1}$, $\mathop c\limits^{ \to }_{2}$) being the basis vector of the primitive cell, and ($h_{1}$, $h_{2}$) the integers. Similarly, $m_{1} \mathop a\limits^{ \to }_{1} + m_{2} \mathop a\limits^{ \to }_{2}$ and $n_{1} \mathop b\limits^{ \to }_{1} + n_{2} \mathop b\limits^{ \to }_{2}$ are the basis vectors of MoS_2_ and graphene supercells, respectively, with ($\mathop a\limits^{ \to }_{1}$, $\mathop a\limits^{ \to }_{2}$) and ($\mathop b\limits^{ \to }_{1}$, $\mathop b\limits^{ \to }_{2}$) being the basis vectors of the the primitive cells, and as ($m_{1}$, $m_{2}$) and ($n_{1}$, $n_{2}$) are integers. As set of values of $m_{1}$, $m_{2}$, $n_{1}$, and $n_{2}$ ($h_{1}$, and $h_{2}$) are determined so that the lattice mismatch $\delta_{1}$ ($\delta_{2}$) between the supercells of MoS_2_ and graphene (metal) meets the following condition:

1 $$- \delta_{1} \le \frac{{\left| {n_{1} \mathop b\limits^{ \to }_{1} + n_{2} \mathop b\limits^{ \to }_{2} } \right| - \left| {m_{1} \mathop a\limits^{ \to }_{1} + m_{2} \mathop a\limits^{ \to }_{2} } \right|}}{{\left| {m_{1} \mathop a\limits^{ \to }_{1} + m_{2} \mathop a\limits^{ \to }_{2} } \right|}} \le \delta_{1}$$

2 $$- \delta_{2} \le \frac{{\left| {h_{1} \mathop c\limits^{ \to }_{1} + h_{2} \mathop c\limits^{ \to }_{2} } \right| - \left| {m_{1} \mathop a\limits^{ \to }_{1} + m_{2} \mathop a\limits^{ \to }_{2} } \right|}}{{\left| {m_{1} \mathop a\limits^{ \to }_{1} + m_{2} \mathop a\limits^{ \to }_{2} } \right|}} \le \delta_{2}$$

The $\sqrt N \times \sqrt N$, and $\sqrt H \times \sqrt H$ unit cells of graphene and metal are adjusted to the $\sqrt M \times \sqrt M$ unit cell of MoS_2_, in which $N = n_{1}^{2} + n_{2}^{2} + n_{1} n_{2}$, $H = h_{1}^{2} + h_{2}^{2} + h_{1} h_{2}$, and $M = m_{1}^{2} + m_{2}^{2} + m_{1} m_{2}$. For the in-plane lattice mismatch within $\delta_{1}$ and $\delta_{2}$ ($\le 5\%$), the supercells of the contacts are usually larger with a broken symmetry. Based on this approach, the CSL of MoS_2_, graphene, and metal are modeled, and the parameters are listed in Table 1. We adopt $3 \times 3$ MoS_2_ and $4 \times 4$ graphene supercells to $3 \times 3$ Mg(0001) and Cu(111) supercells, $4 \times 4$ MoS_2_ and $5 \times 5$ graphene supercells to $4 \times 4$ In(101) supercell, $2\sqrt 3 \times 2\sqrt 3$ MoS_2_ and $\sqrt {19} \times \sqrt {19}$ graphene supercells to Al(0001) and Pd(111) supercells, and $2\sqrt 3 \times 2\sqrt 3$ Sc(0001) and Ti(0001) supercells, and $\sqrt {13} \times \sqrt {13}$ MoS_2_ and $\sqrt {19} \times \sqrt {19}$ graphene supercells to $4 \times 4$ Ag(111) and Au(111) supercells, respectively. As listed in Table 1, the lattice constant mismatches are all less than 5%. Similarly, the MoS_2_/metal contacts by inserting bi-layer graphene are also constructed and as shown in Fig. 1d.

**Table 1:** In-plane supercell defined by the MoS_2_ basis vector $m_{1} \mathop a\limits^{ \to }_{1} + m_{2} \mathop a\limits^{ \to }_{2}$, graphene basis vector $n_{1} \mathop b\limits^{ \to }_{1} + n_{2} \mathop b\limits^{ \to }_{2}$, and the metal basis vector $h_{1} \mathop c\limits^{ \to }_{1} + h_{2} \mathop c\limits^{ \to }_{2}$ for MoS_2_/metal with inserting single-layer graphene.

Metal	*m*_1_, *m*_2_	*n*_1_, *n*_2_	*h*_1_, *h*_2_	*δ*_1_ (%)	*δ*_2_ (%)
Mg	3, 0	4, 0	3, 0	3.8	1
Al	2, 2	3, 2	4, 0	0.8	1.09
In	4, 0	5, 0	4, 0	2.7	4.15
Cu	3, 0	4, 0	3, 0	3.8	0.73
Ag	4, − 1	5, − 2	4, 0	1.9	0.93
Au	4, − 1	5, − 2	4, 0	1.9	0.81
Pd	2, 2	3, 2	4, 0	0.8	0.34
Sc	2, 2	3, 2	2, 2	0.8	3
Ti	2, 2	3, 2	2, 2	0.8	4.74

$\delta_{1}$ ($\delta_{2}$) represents the mismatch between the MoS_2_ and graphene (metal) lattices.

The optimized parameters of MoS_2_/metal contact by inserting single- and bi-layer graphene are listed in Table 2. For Mg, Al, In, Cu, Ag, Au, and Pd, the the average vertical separations between the metal and graphene (d_M-G_) are 3.412 Å, 3.167 Å, 3.257 Å, 2.999 Å, 2.828 Å, 3.090 Å, and 2.789 Å, respectively, and they are longer than the covalent bond lengths of Mg–C (2.172 Å), Al–C (1.972 Å), In–C (2.182 Å), Cu–C (2.082 Å), Ag–C (2.212 Å), Au–C (2.122 Å), Pd–C (2.152 Å) pairs, respectively, indicating weak interaction between them.^39^ For Sc and Ti, the values of d_M-G_ are 2.171 Å and 2.112 Å, respectively, and they are shorter than the covalent bond lengths of Sc-C (2.462 Å) and Ti–C (2.362 Å), respectively, indicating strong interaction between them. The values of d_G-G_ are 3.197 Å, 3.042 Å, 3.141 Å, 3.161 Å, 3.272 Å, 3.298 Å, 3.210 Å, and 3.050 Å for Mg, Al, In, Cu, Ag, Au, Pd, Sc, and Ti, respectively, and they are slightly longer than that of the bi-layer graphene (3.355 Å)^40^. Furthermore, the d_G-MS_ of single- or bi-layer graphene insertion are comparable with that of MoS_2_/graphene (3.320 Å), due to the weak van der Waal interaction between them^41^.

**Table 2:** Calculated interfacial properties of MoS_2_/metals by inserting single-layer and bi-layer graphene.

Metal	W_M_ (eV)	Single-layer graphene inserted			Bi-layer graphene inserted			
		d_M-G_ (Å)	d_G-MS_ (Å)	W_M/G_ (eV)	d_M-G_ (Å)	d_G-G_ (Å)	d_G-MS_ (Å)	W_M-2G_ (Å)
Mg	4.321 3.66^b^	3.412	3.341	3.579	3.393	3.197	3.339	3.933
Al	3.878	3.167	3.439	3.662 4.04^a^	3.354	3.042	3.349	4.213
In	3.707	3.257	3.298	3.376	3.236	3.141	3.304	3.812
Cu	4.796 4.65^b^	2.999	3.346	4.324 4.40^a^	3.075	3.161	3.357	4.345
Ag	4.321 4.26^b^	2.828	3.486	4.013 4.24^a^	2.858	3.272	3.349	3.781
Au	4.925 5.10^b^	3.090	3.378	4.566 4.74^a^	2.962	3.298	3.345	4.818
Pd	5.075 5.12^b^	2.789	3.425	4.669 4.03^a^	2.787	3.298	3.338	4.160
Sc	3.276 3.50^b^	2.171	3.246	3.604	2.188	3.210	3.369	3.929
Ti	4.266 4.33^b^ 4.58^c^	2.112	3.243	3.593 4.17^a^	1.952	3.050	3.419	4.053

d_M-G_, d_G-G_, and d_G-MS_, as marked in Fig. 1c,d, are the average vertical separation between the metal and graphene, between bi-layer graphene, and between graphene and MoS_2_, respectively. W_M_ and W_M/G_ (W_M/2G_) are the work functions of the free-standing metal surfaces and adsorbed single-layer (bi-layer) graphene, respectively.

^a^Reference^28^.

^b^Reference^37^.

^c^Reference^38^.

**Figure 2:**
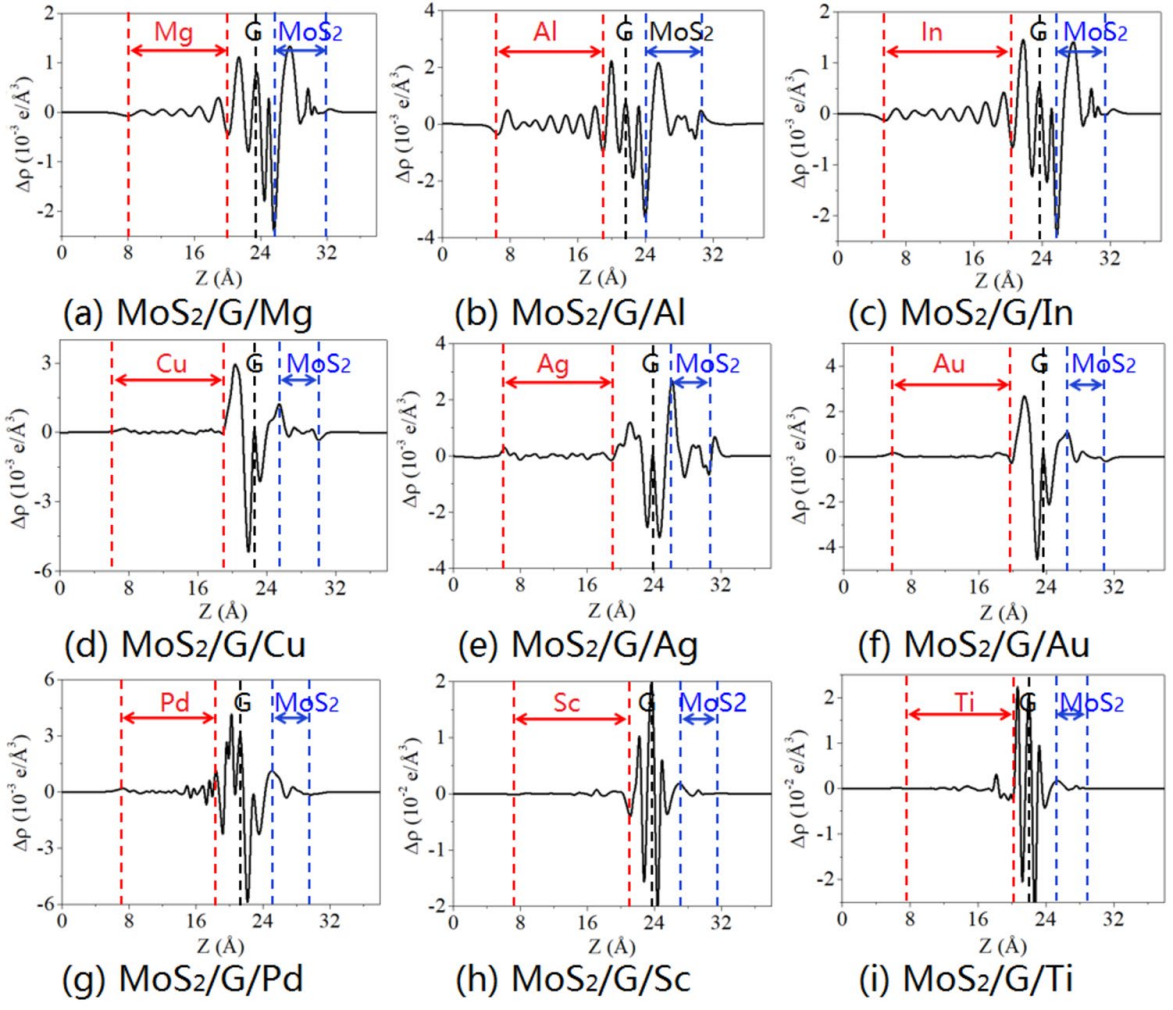
Plane-average charge density difference along the z-direction of MoS_2_/metals by inserting single-layer graphene.

**Figure 3:**
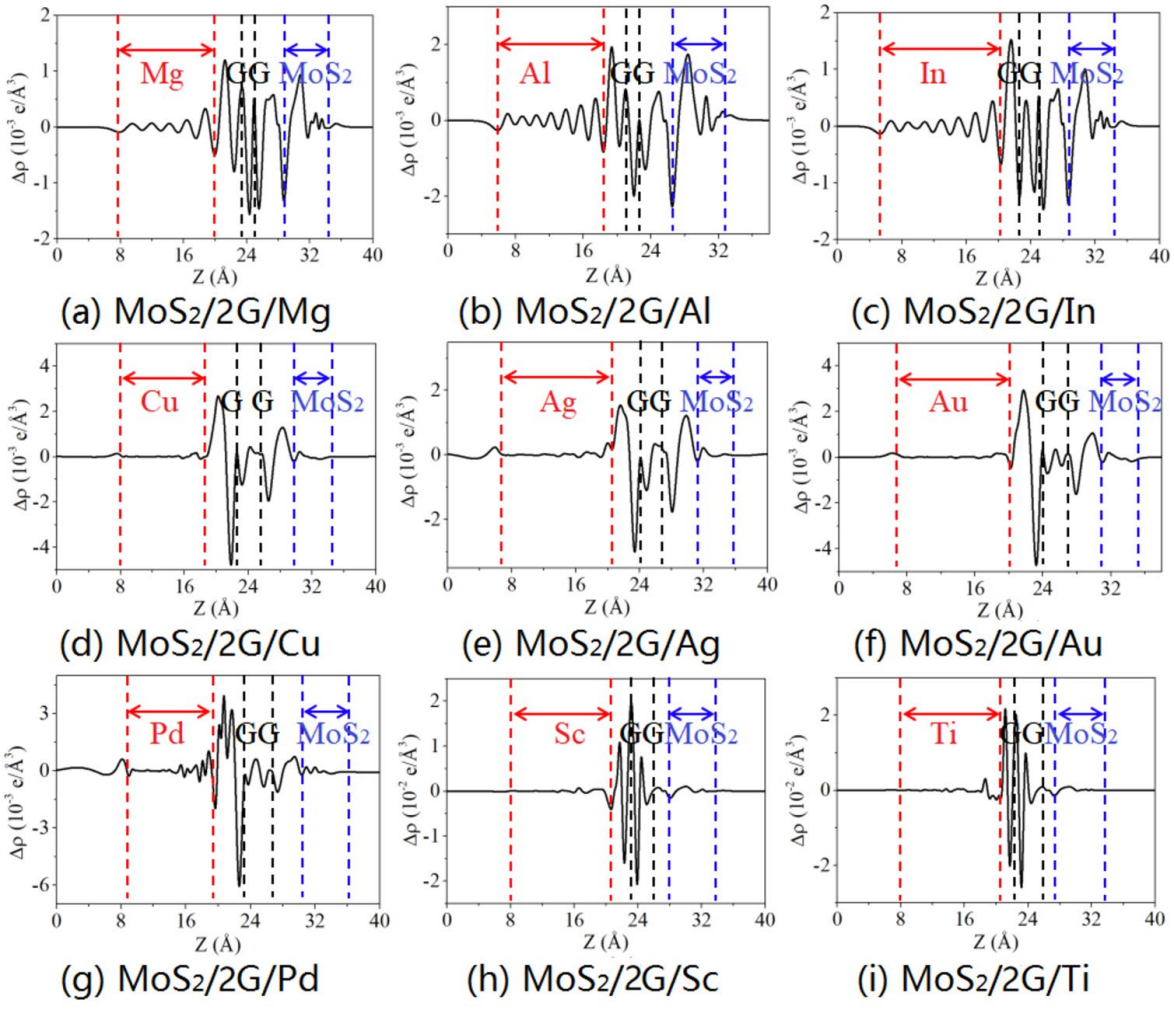
Plane-average charge density difference along the z-direction of MoS_2_/metals by inserting bi-layer graphene.

Figures 2 and 3 display the charge density difference of MoS_2_/metal by inserting single- and bi-layer graphene, respectively. The charge density difference is calculated as:

3 $$\Delta \rho (z) = \int {\rho_{{{\text{contact}}}} } {\text{d}}x{\text{d}}y - \int {\rho_{{{\text{MS}}}} {\text{d}}x{\text{d}}y - \int {\rho_{{\text{i}}} } } {\text{d}}x{\text{d}}y - \int {\rho_{{\text{M}}} } {\text{d}}x{\text{d}}y,$$

in which $\rho_{{{\text{contact}}}}$, $\rho_{{{\text{MS}}}}$, $\rho_{{\text{i}}}$, and $\rho_{{\text{M}}}$ are the charge densities of the contact system, the isolated MoS_2_, inserting layer (single- or bi-layer graphene), and metal, respectively. As shown in Figs. 2 and 3, the charge transfer mainly occurs at the interface between the graphene and metal layers, with smaller transfer at the interface between bi-layer garphene or between graphene and MoS_2_. Specifically, the charge transfer oscillation occurs near the interface between graphene and Sc/Ti, indicating strong interaction and the formation of interfacial dipole layers. Furthermore, the Bader charge analysis has also been conducted for those interfaces. The Bader charge distribution of MoS_2_/metal contact by inserting single-layer graphene exhibits the average charge values of 0.035 *e*, 0.052 *e* and 0.049 *e* at the interface of the graphene/Mg, Al, and In, respectively, showing weak interactions. Medium interaction are observed at the interface of graphene/Cu, Ag, and Au with the average charge values of 0.069 *e*, 0.070 *e* and 0.073 *e*, respectively. The average charge values of 0.101 *e*, 0.125 *e* and 0.208 *e* at the interface between the graphene and Pd, Sc, and Ti, respectively, indicating strong interactions between them. However, the average charge values of 0.032 *e*, 0.042 *e*, 0.034 *e*, 0.038 *e*, 0.042 *e*, 0.042 *e*, 0.046 *e*, 0.039 *e*, and 0.042 *e* at the interface between the graphene and MoS_2_, respectively, indicating weak interactions between them. For MoS_2_/metal contact by inserting bi-layer graphene, the interactions between metal and graphene, as well as between graphene and MoS_2_ are similar to that of the MoS_2_/metal contact by inserting singly-layer graphene.

**Figure 4:**
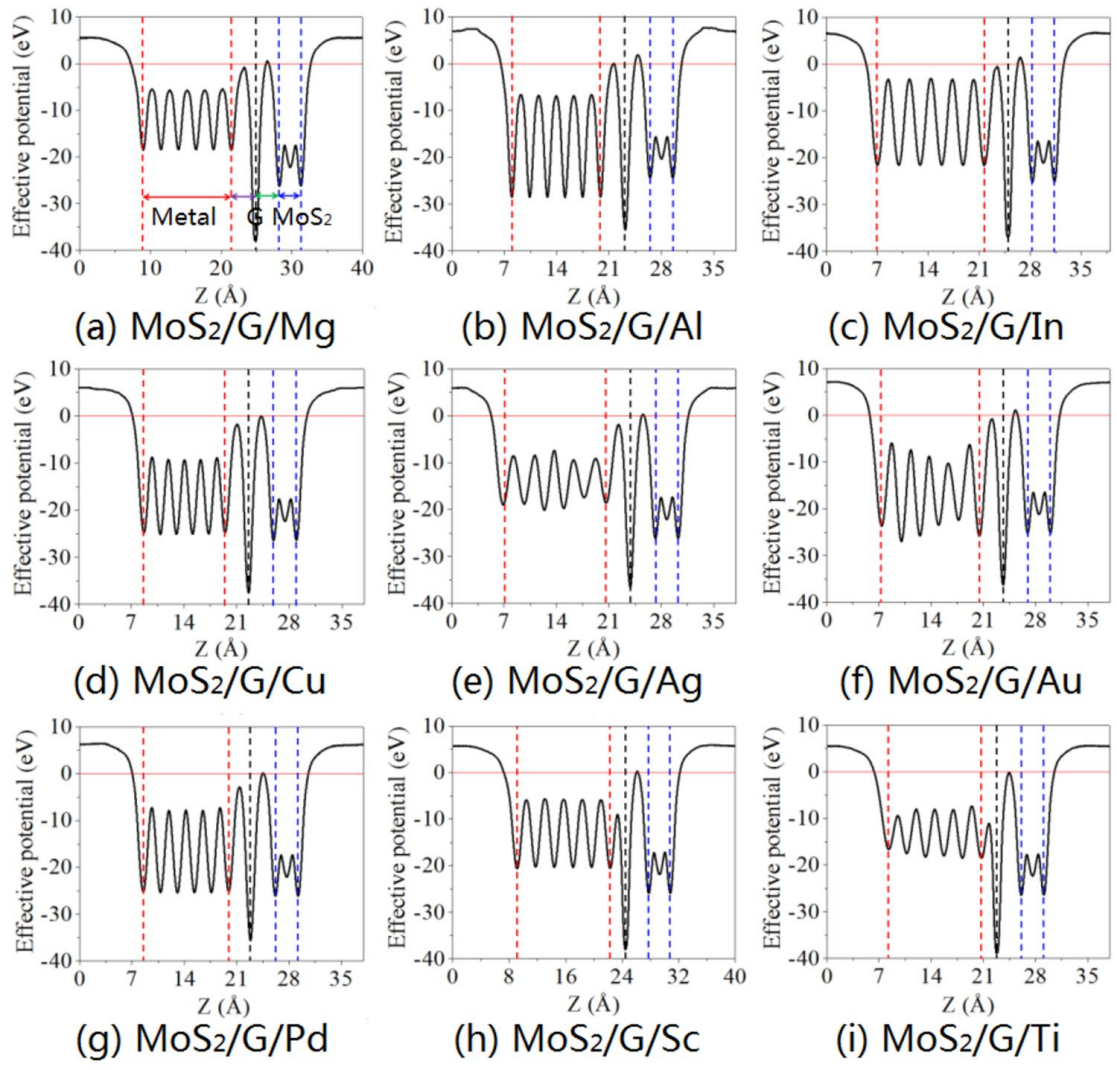
Plane-average electronic potential along the z-direction of MoS_2_/metals by inserting single-layer graphene.

**Figure 5:**
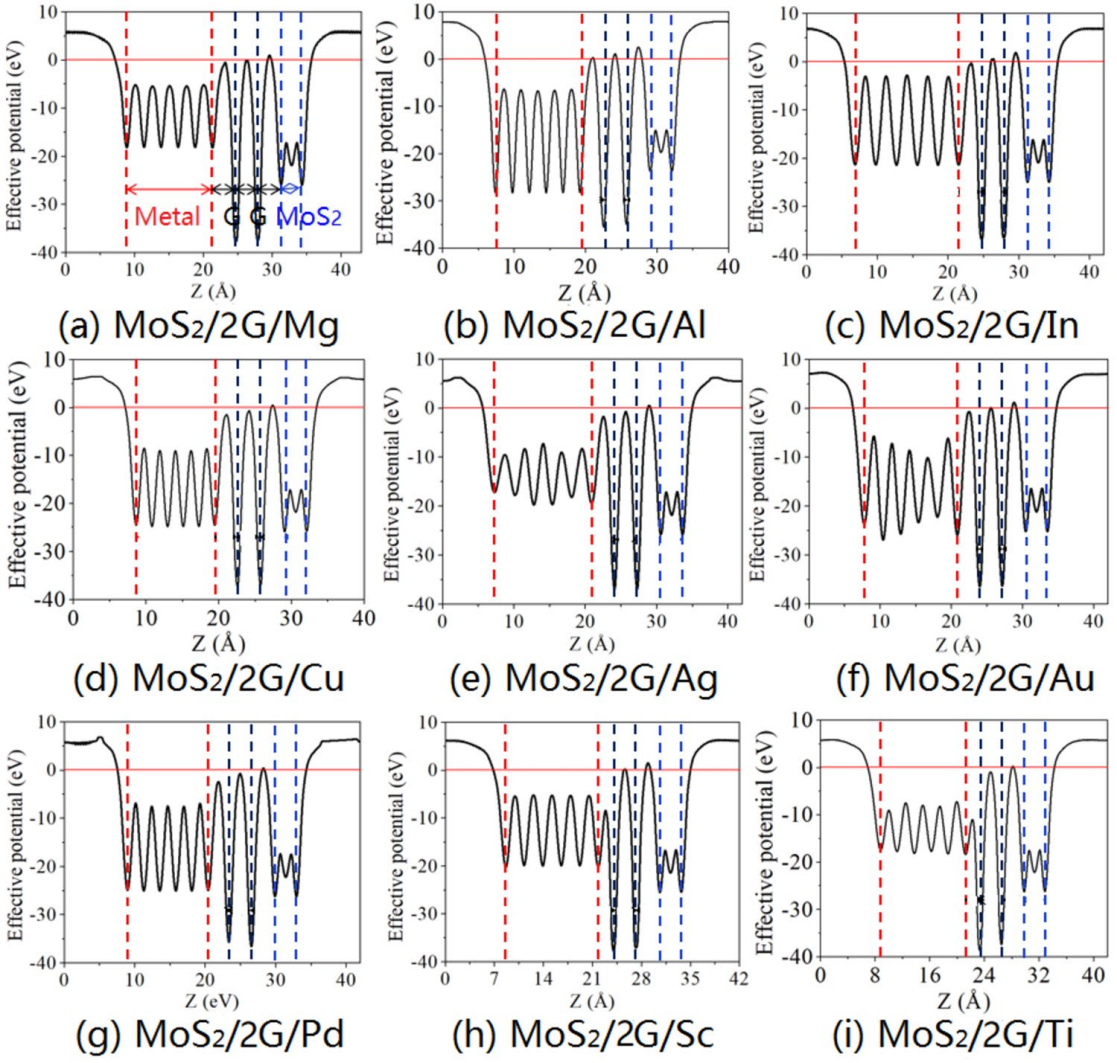
Plane-average electronic potential along the z-direction of MoS_2_/metals by inserting bi-layer graphene.

To investigate the effects of the inserting singly-layer and bi-layer graphene on the electron tunnel of MoS_2_/metal contact, the average effective potential in the x–y plane normal to the interface and the tunneling barrier $\Delta V$ are calculated, as shown in Figs. 4 and 5, respectively. The height $\Delta V$ defined as the potential energy above $E_{{\text{F}}}$ between graphene and metal, bi-layer graphene, as well as garphene and MoS_2_, and its width $\omega_{{\text{B}}}$ is defined as the full width at half maximum of $\Delta V$. The barrier height reflects the lowest barrier that electrons at $E_{{\text{F}}}$ need to overcome upon injection between neighboring layers. With singly-layer graphene, no tunneling barrier exists at the graphene/metal interface. However, there is noticeable tunneling barrier at exists at the graphene/MoS_2_ interface with the values of $\Delta V$ are 0.568 eV, 1.889 eV, 1.456 eV, 0.309 eV, 1.061 eV, 0.111 eV and 0.313. The corresponding values of $\omega_{{\text{B}}}$ are 0.422 Å, 0.826 Å, 0.654 Å, 0.292 Å, 0.562 Å, 0.153 Å, and 0.252 Å for Mg, Al, In, Ag, Au, Pd, and Sc, respectively (excluding Cu and Ti). For inserting bi-layer graphene, the tunneling barrier only exists at the interface between graphene and Al, and the values of $\Delta V$ and $\omega_{{\text{B}}}$ are 0.420 eV and 0.164 Å, respectively; At the interface between bi-layer graphene for Al, In, Au, and Sc, the values of $\Delta V$ are 1.320 eV, 0.620 eV, 0.181 eV, and 0.296 eV, as well as the values of $\omega_{{\text{B}}}$ are 0.281 Å, 0.233 Å, 0.246 Å, and 0.192 Å, respectively; At the interface between graphene and MoS_2_ for Al, Mg, In, Cu, Ag, Au, Pd, Sc, and Ti, the values of $\Delta V$ are 1.070 eV, 2.604 eV, 1.990 eV, 0.566 eV, 0.586 eV, 1.270 eV, 0.466 eV, 1.590 eV and 0.360 eV, as well as the values of $\omega_{{\text{B}}}$ are 0.389 Å, 0.398 Å, 0.240 Å, 0.179 Å, 0.308 Å, 0.330 Å, 0.299 Å, 0.380 Å and 0.184 Å, respectively. The tunneling probability $T_{{\text{B}}}$ can be evaluated as follows:

4 $$T_{{\text{B}}} = \exp \left( { - 2 \times \frac{{\sqrt {2m\Delta V} }}{\hbar } \times \omega_{{\text{B}}} } \right),$$

where $m$ is the effective mass of a free electron and $\hbar$ is the Planck’s constant. The $T_{{\text{B}}}$ values at the interface between graphene and MoS_2_ in the MoS_2_/metal contact by inserting singly-layer graphene are estimated to be 88%, 62.2%, 69.7%, 93.1%, 77.4%, 97.8%, and 94% for MoS_2_ contacted with Mg, Al, In, Ag, Au, Pd, and Sc, respectively. For MoS_2_/metal contact with bi-layer graphene insertion, the $T_{{\text{B}}}$ values at the interface between graphene and Al are estimated to be 96.5%; The $T_{{\text{B}}}$ values at the interface between bi-layer graphene are estimated to be 89.7%, 94.0%, 96.5% and 96.5% for Al, In, Au, and Sc, respectively; The $T_{{\text{B}}}$ values at the interface between graphene and MoS_2_ are estimated to be 87.4%, 80.5%, 89.2%, 95.6%, 92.4%, 88.2%, 93.4%, 85.1%, and 96.4% for Mg, Al, In, Cu, Ag, Au, Pd, Sc, and Ti, respectively. Therefore, inserting graphene is not determinant to the tunneling transmission in MoS_2_/metal contacts.

**Figure 6:**
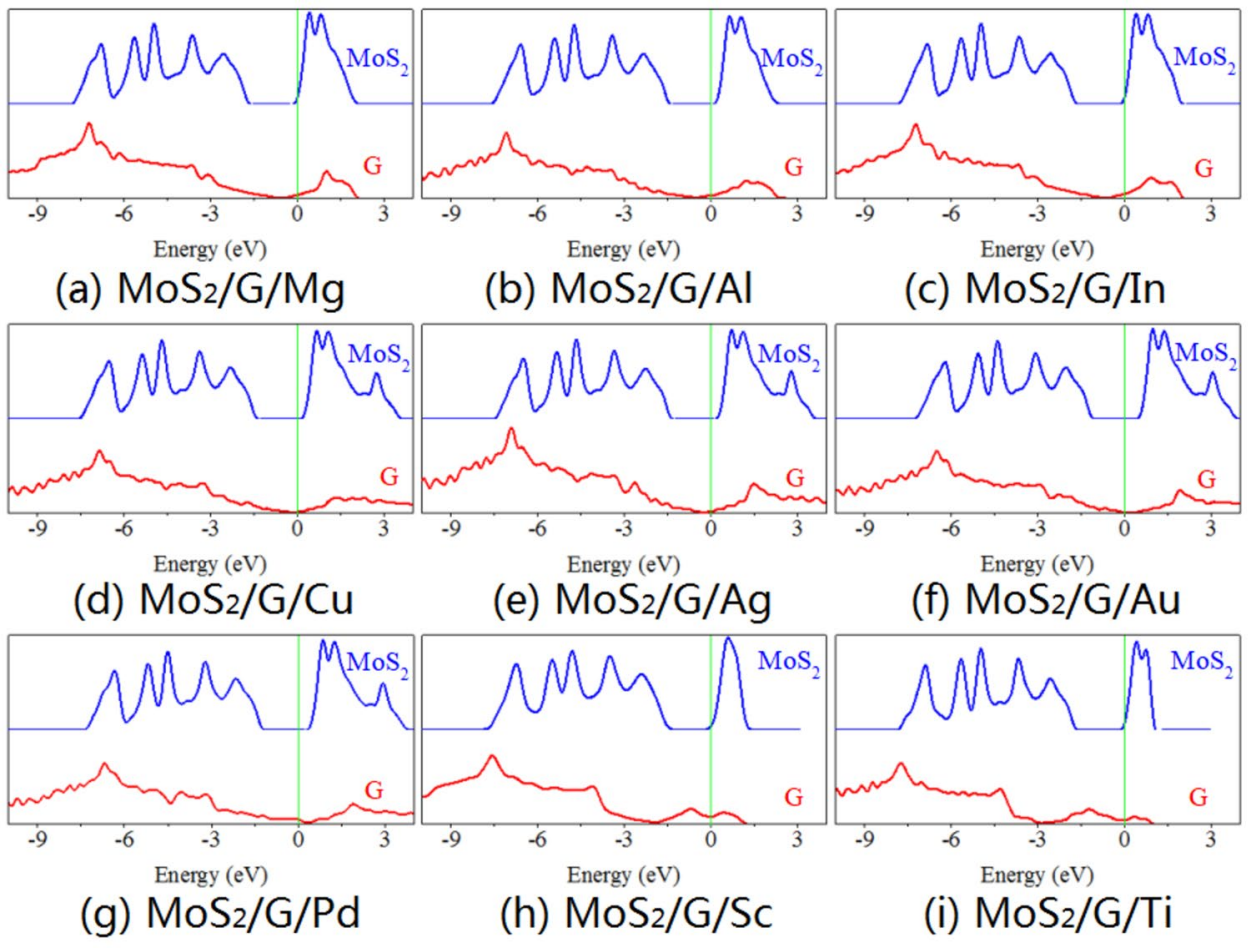
Partial density of states (PDOS) of graphene and MoS_2_ layers in MoS_2_/metals by inserting single-layer graphene.

**Figure 7:**
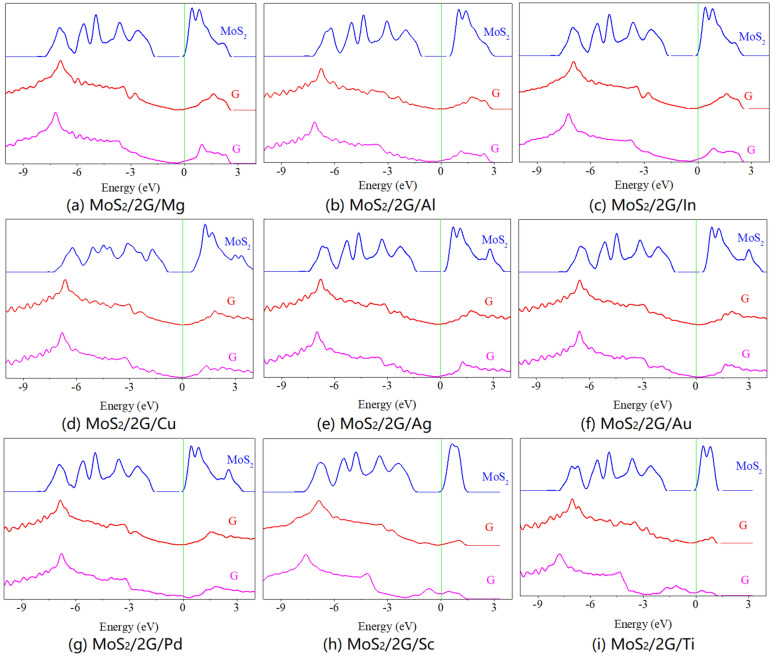
Partial density of states (PDOS) of graphene and MoS_2_ layers in MoS_2_/metals by inserting bi-layer graphene.

As shown in Figs. 6 and 7, the partial density of states (PDOS) of graphene and MoS_2_ sublayers in MoS_2_/metal contacts by inserting single- and bi-layer graphene has been studied, respectively. While graphene does not introduce an additional contact barrier, it induces significant change of electronic structure on itself. For the MoS_2_/metal contact by inserting singly-layer graphene, the Dirac cone gets shifted and below Fermi level for Mg, Al, In, Cu, and Ag, but exhibits opposite trend for Au. Moreover, the Dirac nature is completely lost when MoS_2_ contacted with Pd, Sc, and Ti. The same phenomenon occurs for graphene which is adjacent to metal layer in the MoS_2_/metal contact by inserting bi-layer graphene, but the Dirac cone is not perturbed for graphene which is adjacent to MoS_2_ layer. Compared to the free monolayer MoS_2_, although the semiconductor future is maintained, the energy band alignment and band gap of the MoS_2_ in MoS_2_/metal by inserting single- and bi-layer graphene have changed. For inserting singly-layer graphene, the conduction band of MoS_2_ across Fermi level when contacted with Mg, In, Sc, and Ti, while the Fermi level lies in the band gap and closes to the conduction band minimum of MoS_2_ when contacted with Al, Cu, Ag, Au, and Pd, indicating an n-type semiconductor. The values of band gap are 1.567 eV, 1.526 eV, 1.540 eV, 1.562 eV, 1.562 eV, 1.553 eV, 1.536 eV, 1.482 eV, and 1.540 eV for MoS_2_ when contacted with Mg, Al, In, Cu, Ag, Au, Pd, Sc, and Ti, respectively. The same phenomenon occurs for inserting bi-layer graphene except for MoS_2_ contacted with Pd. And the values of band gap are 1.572 eV, 1.589 eV, 1.544 eV, 1.300 eV, 1.571 eV, 1.544 eV, 1.544 eV, 1.466 eV and 1.593 eV for MoS_2_ when contacted with Mg, Al, In, Cu, Ag, Au, Pd, Sc, and Ti, respectively. This again implies that graphene is successful in isolating the MoS_2_ layer from metal with equivalent zero midgap states and only shifts of valence band maximum and conduction band minimum with respect to free MoS_2_, and the interaction between graphene and metal dictates the amount and nature of shift.

**Figure 8:**
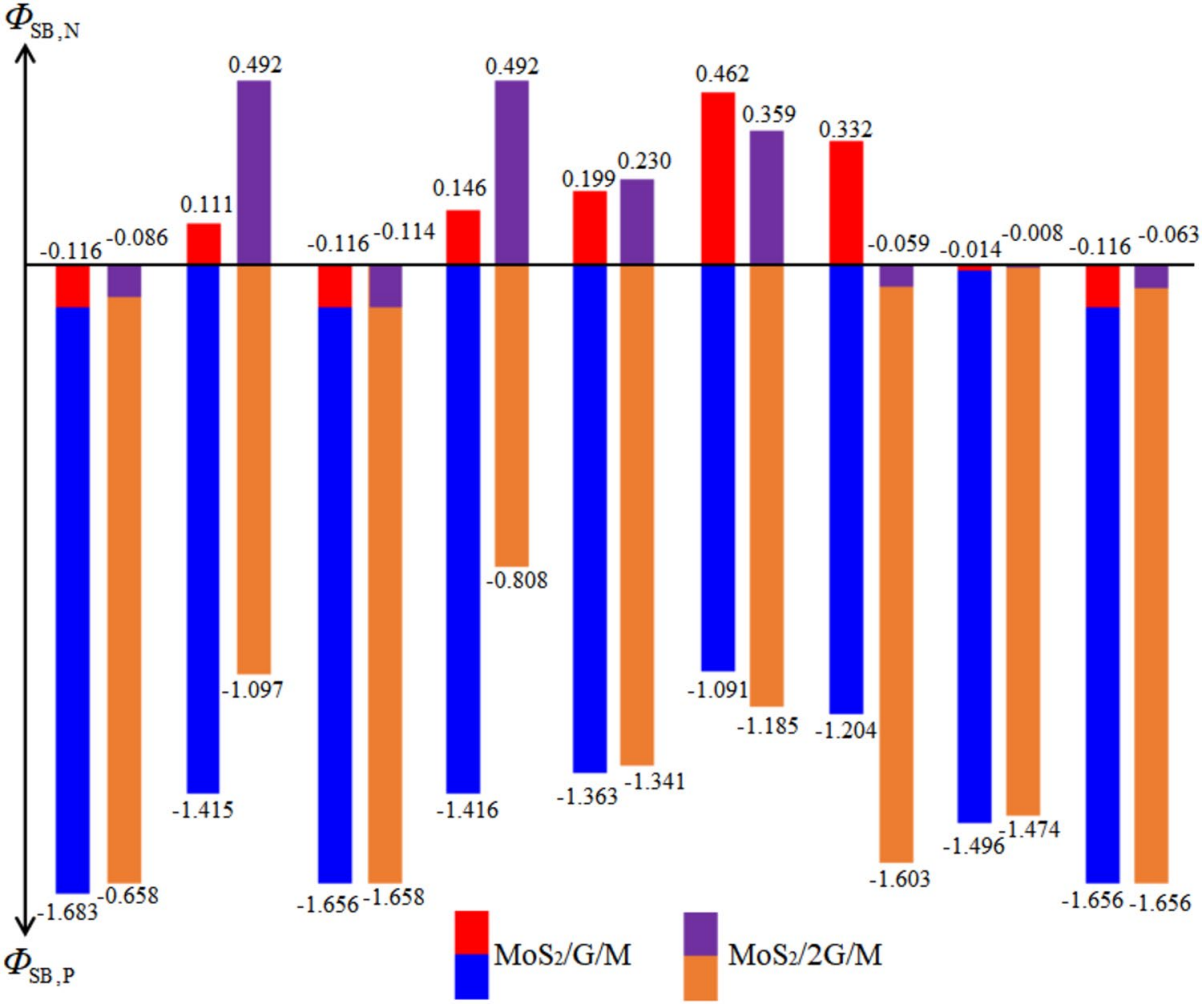
Schottky barrier height ($\Phi_{{{\text{SB}}}}$) of MoS_2_/metals by inserting single-layer and bi-layer graphene.

Finally, the Schottky barrier height $\Phi_{{{\text{SB}}}}$ has been calculated and summarized in Fig. 8. The barrier height $\Phi_{{\text{SB,N}}}$ are 0.111 eV, 0.146 eV, 0.199 eV, 0.462 eV, and 0.332 eV for MoS_2_ contacted with Al, Cu, Ag, Au, and Pd by inserting single-layer graphene, respectively. Conversely, when MoS_2_ is contacting with Mg, In, Sc, and Ti with single-layer graphene insertion, the negative n-type Schottky barrier is formed with the $\Phi_{{\text{SB,N}}}$ of − 0.116 eV, − 0.116 eV, − 0.014 eV, and − 0.116 eV, respectively, indicating an ohmic contact. For bi-layer graphene insertion, the barrier height $\Phi_{{\text{SB,N}}}$ of MoS_2_ contacted with Al, Cu, Ag, and Au are 0.492 eV, 0.492 eV, 0.230 eV, and 0.359 eV, respectively. When MoS_2_ contacted with Mg, In, Pd, Sc, and Ti with bi-layer graphene insertion, the negative n-type Schottky barrier is formed with the $\Phi_{{\text{SB,N}}}$ of − 0.086 eV, − 0.114 eV, − 0.059 eV, − 0.008 eV, and − 0.0636 eV, respectively.

## Conclusions

In this study, the effects of inserting single- and bi-layer graphene on the electronic properties and Schottky barrier of MoS_2_/metals (Mg, Al, In, Cu, Ag, Au, Pd, Ti, and Sc) are studies by using first-principle calculations based on density functional theory. Our findings indicate significant charge value at the interface between graphene and metals, leading to the absence of tunneling barrier appears in the MoS_2_/metal contact by inserting singly-layer graphene. By contrast, the tunneling barrier exists at the interface between graphene and MoS_2_, suggests hindering in electron injection. Additionally, the $\Phi_{{\text{SB,N}}}$ is reduced when graphene is inserted in MoS_2_/metal contacts. When MoS_2_ contacted with Mg, In, Sc, and Ti by inserting single-layer graphene, the $\Phi_{{\text{SB,N}}}$ of − 0.116 eV, − 0.116 eV, − 0.014 eV, and − 0.116 eV, respectively. On the other hand, bi-layer graphene insertion leads to the negative n-type Schottky barriers of − 0.086 eV, − 0.114 eV, − 0.059 eV, − 0.008 eV, and − 0.0636 eV for MoS_2_ contacted with Mg, In, Pd, Sc, and Ti, respectively, indicating transition to the Ohmic contact. Our findings offer valuable insights for the design and optimization of nanoelectronic devices, utilizing MoS_2_/graphene/metal interfaces, highlighting the potential for enhanced device performance through graphene insertion.

## Data Availability

The datasets used and/or analysed during the current study available from the corresponding author on reasonable request.
